# Preoperative Anxiety in Candidates for Heart Surgery

**Published:** 2014

**Authors:** Mehdi Fathi, Seyed Mostafa Alavi, Marjan Joudi, Mitra Joudi, Helia Mahdikhani, Rasool Ferasatkish, Houman Bakhshandeh, Mehdi Jabbari Nooghabi

**Affiliations:** 1 Assistant Professor, Surgical Oncology Research Center, Imam Reza Hospital AND Department of Anesthesiology, School of Medicine, Mashhad University of Medical Sciences, Mashhad, Iran.; 2 Associate Professor, Department of Anesthesiology, School of Medicine, Tehran University of Medical Sciences, Tehran, Iran.; 3 Assistant Professor, Surgical Oncology Research Center, Imam Reza Hospital AND Department of Surgery, School of Medicine, Mashhad University of Medical Sciences, Mashhad, Iran.; 4 Assistant Professor, Department of Psychiatry, School of Medicine, Gorgan University of Medical Sciences, Gorgan, Iran.; 5 Psychologist, Department of Psychology, Islamic Azad University, Tehran Branch, Tehran, Iran.; 6 Professor, Department of Anesthesiology, School of Medicine, Tehran University of Medical Sciences, Tehran, Iran.; 7 Epidemiologist, Shahid Rajaee Heart Center, Tehran, Iran.; 8 Assistant Professor, Department of Statistics, School of Mathematical Sciences, Ferdowsi University of Mashhad, Mashhad, Iran.

**Keywords:** Anxiety, Heart Surgery, State-Trait Anxiety Inventory (STAI)

## Abstract

**Objective:** The goal of this study was to evaluate preoperative anxiety and its predisposing factors in a group of adult patients who were candidate for any kind of heart surgery.

**Methods:** We evaluated preoperative anxiety in 300 patients undergoing heart surgery whose ages ranged between 18-65 years. Relationship of probable demographic factors like gender, educational level, marital status, number of children, family support, opium addiction, occupational status, and left ventricular ejection fraction (LVEF) with anxiety level of the patients were evaluated. To determine anxiety, the State-Trait Anxiety Inventory (STAI) was completed by the subjects.

**Results: **Descriptive anxiety levels showed that mean of state and trait anxiety of our studied patients were in moderate scaling. Correlation between state and trait anxiety was more prominent in females (r = 0.80) than in males (r = 0.70) (p < 0.001). Distribution of males and females was significantly different (p = 0.048). All the patients had significantly different underlying diseases (p = 0.018), opioid addiction (yes/no) was significantly different in all of the patients (p < 0.001), while family support (yes/no) was not significantly different in all of the patients (p = 0.453). There were significant differences between mean of preoperative anxiety at different LVEF values of any EF level (p < 0.001, F = 6.47); those who had LVEF of more than 50% had significantly lower mean anxiety scores.

**Conclusion:** Preoperative psychiatric consultation should be focused more on women and patients with higher EF. Moreover, physical activity strength may be effective on reducing preoperative anxiety.

## Introduction

It is widely accepted that preoperative anxiety is a common reaction (1). Anticipation of a necessary surgical intervention induces emotional shock for patients and their family members (2). Spielberger defined anxiety as a negative or threatening emotion that one feels generally, in the long term (trait anxiety), or in a specific situation that fluctuates over time (state anxiety). Anxiety in patients could be induced by unfamiliar situations, physical separation from family, no or little knowledge about medical interventions, diagnosis, or therapeutic methods, and high costs of operation, hospitalization, anesthesia induction, post-operative pain, possible future disabilities, and death (3). The prevalence of anxiety in highly-selective patients prepared for elective operations has been reported as high as 80% (4, 5).

Anxiety during surgery might influence the desired outcomes of surgical treatment and after surgery affect the predicted clinical improvement and mental health. Moreover, anxiety causes hypertension, increased heart rate, and it might lead to bleeding and other probable post-operation side effects (5). On the other hand, long-term anxiety increases metabolism, oxygen consumption, and emotional conception of pain. 

The aim of this study was to evaluate preoperative anxiety in patients who were candidates for open heart surgery. Moreover, we investigated the relation of probable demographic factors with anxiety levels.

## Materials and Methods

This analytical descriptive study was carried out among 300 patients who were candidates for open heart surgery of any kind and were admitted to the Heart Surgical Ward of Shahid Rajaee Heart Hospital, Tehran, Iran, from June 2010 to November 2010. The age range was 18-65 years. 168 (56%) patients were women and 132 (44%) were men. State anxiety and trait anxiety were measured by the Persian version of the State-Trait Anxiety Inventory (STAI) with proven validity of 91.635 using Cronbach's alpha (6). 

*Data gathering*

The patients were asked to give their demographic information and complete the STAI (1) for evaluating anxiety levels the night before heart surgery at the bedside. The checklist applied included demographic information which consisted of age, gender, educational level, marital status, number of children, family support, opioid addiction, and occupational status. Left ventricular ejection fraction (LVEF) was recorded by ultrasound echocardiography.

The STAI has two sections. The first part assesses situational anxiety (clear or state) and contains 20 questions with four-point scales including “no”, “not really”, “on the whole, yes”, and “yes”. The total score ranges from 20 to 80. The second section questions stable anxiety (hidden or trait) and includes 20 questions with 4-point scales including “seldom or never”, “sometimes”, “often”, and “almost always”. In this inventory we divided the questions into positive and negative scales in order to receive more accurate answers and we pointed reverse scale answers to question numbers 1, 2, 5, 8, 10, 11, 15, 16, 19, 20, 21, 23, 26, 27, 30, 33, 34, 36, and 39. 

We used the New York Heart Association Functional Class (NYHA-FC) to assess physical activity strength. It provides a simple way of classifying the extent of heart failure. It places patients in one of four categories based on how much they are limited during physical activity; the limitations/symptoms are in regards to normal breathing, and varying degrees of shortness of breath and or angina pain.

All questionnaires were completed by a psychologist during conversation with patients. Finally, we categorized the gathered data of each section for state and trait anxiety into mild (scale: 20-39), moderate (scale: 40-59), and sever anxiety (scale: 60-80). 

The medical ethics committee of our university approved the study and all the patients' information was kept confidential. An informed written consent was obtained from every participant. Furthermore, the researcher undertook the responsibility of any probable damage threatening the patients.

*Statistical analysis*

Data analyses were performed using the SPSS software (version 11.5; SPSS Inc., Chicago, IL., USA). Numerical data are expressed as mean (± SD) or percent as proportions to the sample size. We performed Student’s t-test, one way ANOVA (or nonparametric tests such as Mann-Whitney and Kruskal-Wallis), Pearson (or Spearman) correlation coefficient, and linear regression to analyze the qualitative variables. Moreover, we used the chi-square test to compare the distribution of patients in respect to gender, preoperative anxiety, opioid addiction, underlying disease, family support. P-values less than 0.05 were considered statistically significant.

## Results

Regarding the severity of anxiety, it was revealed that means of state and trait anxiety level were in the moderate category, but total anxiety was of the severe category (Table 1). The data showed that each patient was different from others in the aspect of anxiety experience. However, our findings showed both moderate state and trait anxiety levels to be present. Only few patients experienced severe state and trait anxiety (Table 2). Positive correlation of state and trait anxiety was found more common in females than in males (p < 0.001). This positive correlation showed more relation between state and trait anxiety in females (r = 0.80) than in males (r = 0.70). Evaluation of correlation results showed that state, trait, and total anxiety had positive significant correlation with age (p < 0.001; Table 2). We observed higher preoperative anxiety levels in older patients. We evaluated partial regression according to the high correlation observed between anxiety level and age. The results showed that only the correlation between state anxiety and age was significant (p < 0.001), but trait anxiety was independent of age (p = 0.96, r = -0.002).

**Table 1:** Descriptive statistics for preoperative state, and trait and total anxiety in candidates for heart surgery

**Anxiety**	**Number**	**Minimum score**	**Maximum score**	**Mean (± SD)**
**State**	300	20	068	41.60 (± 9.78)
**Trait**	300	20	077	42.14 (± 9.58)
**Total**	300	43	134	84.00 (± 18.24)

Anxiety category scales were as follows: mild (20-39),

moderate (40-59), and sever (60-80).

**Table 2:** Correlation between quantitative anxiety, and age and descriptive statistics for categorical anxiety in candidates for heart surgery

**Quantitative Anxiety**	**Age (Pearson correlation coefficient)**	**Categorical Anxiety**		
		**Mild** **(%)**	**Moderate** **(%)**	**Sever (%)**
**State anxiety**	0.226 p < 0.001	117 (39.0)	172 (57.3)	11 (3.7)
**Trait anxiety**	0.293 p < 0.001	125 (41.7)	167 (55.7)	08 (2.7)

The chi-square test results for the comparison of the frequency distribution of different underlying variables are shown in table 3. Distribution of males and females was significantly different (p = 0.048), all the patients have significantly different underlying diseases (p = 0.018), and opioid addiction (yes/no) was significantly different in all of the patients (p < 0.001). However, family support (yes/no) was not significantly different (p = 0.453).

One way ANOVA demonstrated that our patients experienced different significant preoperative anxiety according to marital status (p < 0.001, F = 8.86), education level (p < 0.001, F = 6.34), occupational situation (p = 0.005, F = 3.17), and physical activity strength (p < 0.001, F = 9.47) (Table 4). As shown in table 4, total mean anxiety score decreased significantly with higher educational levels, and maximum anxiety levels were attributed to other profession groups, being homemaker, and then unemployed patients, respectively.

**Table 3:** Comparison of frequency distribution of gender, underlying diseases, opioid addiction, and familial support of the patients studied

**Variable**		**Number of patients (%)**	**P value**
**Gender**	**Male**	168 (56.0)	0.043
	**Female**	132 (44.0)	
**Underlying diseases**	**No**	171 (57.0)	0.018
	**Yes**	129 (43.0)	
**Opioid addiction**	**No**	284 (94.7)	< 0.001
	**Yes**	16 (5.3)	
**Familial support**	**No**	143 (47.7)	0.453
	**Yes**	157 (52.3)	

In addition, recorded data for patients with different physical activity strength (according to NYHA-FC) demonstrated that in the worse functional class, mean anxiety score increased. 

Most of our studied patients had one (57%) or more (40.33%) children. Analysis of number of children and preoperative anxiety indicated that the number of children was not related to the anxiety level (p = 0.54). 

**Figure 1:**
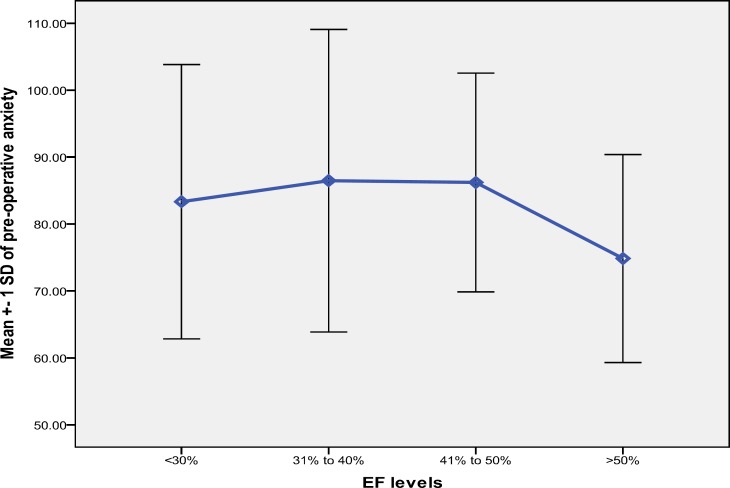
Mean total preoperative anxiety according to different left ventricular ejection fraction values (the differences are significant if p < 0.001, and F = 6.47)

The patients with LVEF levels between 31-50% had increased anxiety score and patients with lower LVEF (< 30%) and higher LVEF levels (> 50%) experienced lower anxiety levels. Ultrasound echocardiography showed that parallel to LVEF increase, preoperative anxiety increased. However, in higher LVEF levels (> 50%), it was significantly decreased (p < 0.001, F = 6.47; Figure 1). Figure 1 shows mean (± SD) of the STAI scores based on different LVEF values. Furthermore, most of our patients had significantly increased anxiety levels due to an increase in their physical activity strength.

## Discussion

Elevated anxiety scores in coronary artery patients have been reported as 20 to 55% (7, 8).

**Table 4:** Mean (± standard deviation) of total preoperative anxiety scores based on marital status, educational status, job status, and physical activity strength (NYHA-FC)

**Marital Status**	**N** ‡	**Mean** **(± SD)**	**Educational Status**	**N** ‡	**Mean** **(± SD)**	**Job Status**	**N** ‡	**Mean** **(± SD)**	**NYHA-FC** †	**N** ‡	**Mean** **(± SD)**
**Single**	43	78.86 (+ 18.94)	Semi-illiterate	50	92.68 (+ 19.34)	Unemployed	18	86.50 (+ 15.46)	1	9	86.00 (+ 26.94)
**Married**	182	81.59 (+ 19.00)	Elementary	115	86.23 (+ 17.63)	Self-employed	28	74.93 (+ 18.63)	2	98	77.66 (+ 19.92)
**Widowed**	57	93.00 (+ 13.72)	High school	84	79.52 (+ 15.59)	Employed	80	81.48 (+ 18.88)	3	154	85.29 (+ 16.39)
**Divorced**	18	92.78 (+ 3.84)	Undergraduate	49	78.35 (+ 19.15)	Employed	16	78.63 (+ 12.27)	4	39	94.72 (+ 11.80)
**Total**	300	84.04 (+ 18.24)	Graduate	2	72.00 (+ 1.41)	Worker	21	81.29 (+ 17.30)	Total	300	84.04 (+ 18.24)
			Total	300	84.04 (+18.24)	Homemaker	101	87.47 (+ 18.40)			
						Other	36	90.03 (+ 16.61)			
						Total	300	84.04 (+ 18.24)			
**P value**	< 0.001			< 0.001			0.005			< 0.001	

†: New York Heart Association functional class;

‡ : Number of patients

The same anxiety levels were found for patients who had underwent coronary artery bypass grafting (CABG) surgery (9). The average patient experienced a medium level of anxiety regarding CABG surgery, with equal and high regression to both state and trait anxiety. Krannich et al. found a negative correlation between age and anxiety before CABG surgery, as younger patients were more anxious than elder patients (10). Decrease in anxiety and depression scores from pre- to post-heart surgery is due to the fact that the patients are under considerable psychological strain before heart surgery. However, they believed this negative correlation to be due to induced thoughts of potential death, as a mentioned possible complication of heart surgery. These thoughts could have been present in the younger and older patients.

Several studies have demonstrated that the candidates for CABG showed high levels of anxiety prior to the surgery, and after the operation their anxiety levels gradually decreased. However a study revealed that pre-operation anxiety of these patients were higher than patients hospitalized for other operations or the public population (4).

Since we did not include a control group from the public population or patients undergoing other operations, we could not make such a comparison. In another study, younger patients showed a higher level of anxiety in comparison to elder patients (11, 8). In the current trial, almost all of the young patients were candidates for non-coronary heart surgeries. However, this group of patients in this study, similar to others, represented high states of anxiety.

In contrast, we found that there was a positive relation between age and state anxiety, but trait anxiety was independent of age. The state anxiety scores of older patients who scheduled for future selective surgery showed a significant correlation with some percent changes in vital signs (blood pressure and heart rate) during anesthetic induction (12).

Widowed or divorced female patients were more prone to experiencing higher stages of anxiety. One study, based on cognitive-behavioral impression approach, was undertaken by nurses on candidates for CABG. It showed that the cardiac rehabilitation and exercise training had not been effective for anxiety, but had reduced depression among these participants (13). Our study did not consider the improvement of cognitive-behavioral function of the patients as a limitation, but it is understood that this intervention can resolve patients’ conflicts. 

The results were the opposite of what we expected. Before the research we believed those who have more physical activity to suffer from less anxiety before the operation, but the results did not support this idea. This outcome may be due to the fact that a person with more physical activity considers more social responsibilities for himself/herself. Therefore, they might be worried if they will be able to do their duties well after the operation or not. Of course, this can be considered as a strong base for future studies. At the moment, we have no strong reasons to prove this dependence, and have found no studies revealing such dependence.

Knopman et al. (14) reported that women and patients with higher trait anxiety were more likely to experience higher levels of state anxiety. Due to the differences in age and co-morbidities, preoperative cognitive impairment might also be more prevalent among women than in men (15). However, investigations of cognitive state in patients before cardiac surgery compared with control patients have included mostly men and younger patients (16, 17).

Our study results confirmed that women experienced more anxiety than men. Higher income rates and better social support may decrease anxiety level (14). Pre-operation health related quality of life seems to be determinant of the state of this index after CABG surgery (18). However, we did not directly evaluate the quality of life level, due to not having known evidence-based criteria for regional social indices. However, we considered the other indirect life quality factors and found that patients with high educational level, agricultural occupation, and self-employed, married or single patients had significantly lower preoperative anxiety levels.

Suitable doctor-patient relationship and anesthesiologists may decrease anxiety levels (19, 20). Previous studies have shown that providing preoperative information to the patients about their surgery methods and procedure decrease patient’s anxiety (21). Findings of the current study revealed that the level of anxiety is not related to familial support or to opium-addiction. Co-morbidities and underlying diseases, and addiction were also related to the level of anxiety. We did not provide any preoperative knowledge about CABG surgery to our patients, but as mentioned above, we observed that higher educated patients presented significantly lower total anxiety scores.

## Conclusion

In conclusion, as trait anxiety is the emotional state related to educational and personal characteristics of people and develops according to patients’ cognitions, one of the routine and common methods for management of this form of anxiety is to change the beliefs and cognitions of the patients. This can be well conducted by psychotherapy and counseling sessions with a psychologist experienced in this area of practice. 

This idea requires further research to encourage the patients undergoing high risk and stressful operations, to participate in psychotherapy sessions prior to the surgery. Moreover, it is recommended, parallel to these sessions for changing patients’ beliefs about the operation and its related risks, that the caregivers provide useful information about the process of the intervention pre- and post-surgery.

## Authors' contributions

MF and SMA conceived and designed the evaluation. MJ participated in designing and drafting parts of the manuscript, and revised the manuscript. MJ and HM have interviewed the patients, evaluated their psychiatric and psychological condition, and completed the questionnaires. RFHB analyzed the clinical and statistical data. MJN reanalyzed, rechecked, and confirmed the statistical data. All authors have read and approved the final manuscript.

## Footenote

**Declaration of interest:** None.
